# Bis[*N*-2-hy­droxy­ethyl,*N*-methyl­dithio­carbamato-κ^2^ *S*,*S*)’-4-{[(pyridin-4-yl­methyl­idene)hydrazinyl­idene}meth­yl]pyridine-κ*N* ^1^)zinc(II): crystal structure and Hirshfeld surface analysis

**DOI:** 10.1107/S2056989017012725

**Published:** 2017-09-15

**Authors:** Grant A. Broker, Mukesh M. Jotani, Edward R. T. Tiekink

**Affiliations:** a 2020 Eldridge Parkway, Apt 1802, Houston, Texas 77077, USA; b Department of Physics, Bhavan’s Sheth R. A. College of Science, Ahmedabad, Gujarat 380001, India; c Research Centre for Crystalline Materials, School of Science and Technology, Sunway University, 47500 Bandar Sunway, Selangor Darul Ehsan, Malaysia

**Keywords:** crystal structure, zinc, di­thio­carbamate, 4-pyridine­aldazine, hydrogen bonding

## Abstract

In the title compound, [Zn(C_4_H_8_NOS_2_)_2_(C_12_H_10_N_4_)], the Zn^II^ atom exists within a NS_4_ donor set defined by two chelating di­thio­carbamate ligands and a pyridyl-*N* atom derived from a terminally bound 4-pyridine­aldazine ligand. The distorted coordination geometry tends towards square-pyramidal with the pyridyl-N atom occupying the apical position. In the crystal, hydroxyl-O—H⋯O(hydrox­yl) and hydroxyl-O—H⋯N(pyrid­yl) hydrogen-bonding give rise to a supra­molecular double-chain along [1-10]; methyl-C—H⋯π(chelate ring) inter­actions help to consolidate the chain. The chains are connected into a three-dimensional architecture *via* pyridyl-C—H⋯O(hydrox­yl) inter­actions. In addition to the contacts mentioned above, the Hirshfeld surface analysis points to the significance of relatively weak π–π inter­actions between pyridyl rings [inter-centroid distance = 3.901 (3) Å].

## Abstract 2

The mononuclear title compound features a Zn^2+^ ion coordinated by two symmetrically binding di­thio­carbamate ligands and one end of a 4-pyridine­aldazine mol­ecule with the resulting NS_4_ donor set tending towards a square-pyramidal geometry.

## Chemical context   

In the realm of coordination polymers/metal–organic framework structures, bridging bipyridyl ligands have proven most effective in connecting metal centres. This is equally true in the construction of coordination polymers of cadmium(II) di­thio­carbamates, Cd(S_2_CN*R*_2_)_2_, *R* = alkyl. Thus, one-dimensional polymers have been found in the crystals of [Cd(S_2_CN*R*_2_)_2_(*NN*)]_*n*_ in cases where *R* = Et and *NN* = 1,2-bis­(4-pyrid­yl)ethyl­ene (Chai *et al.*, 2003 ▸), *R* = Et and *NN* = 1,2-bis­(4-pyrid­yl)ethane (Avila *et al.*, 2006 ▸) and *R* = Benz, *NN* = 4,4′-bipyridyl (Fan *et al.*, 2007 ▸). In an extension of these studies, hydrogen-bonding functionality, in the form of hydroxy­ethyl groups was included in at least one of the *R* groups of Cd(S_2_CN*R*_2_)_2_. It was of some surprise that coord­in­ation polymers based on Cd←N dative bonds were not formed as the putative bridging *NN* ligand was terminally bound. The first example of this phenomenon was noted in a compound closely related to the title compound, *i.e*. Cd[S_2_CN(*n*-Pr)CH_2_CH_2_OH)]_2_(4-pyridine­aldazine)_2_ (Broker & Tiekink, 2011 ▸), for which both potentially bidentate ligands are monodentate. The non-coordinating pyridyl-N atoms participate in hydroxyl-O—H⋯N(pyrid­yl) hydrogen-bonds. In another inter­esting example, regardless of the stoichiometry of the reaction between Cd[S_2_CN(*i*-Pr)CH_2_CH_2_OH]_2_ and 1,2-bis­(4-pyrid­yl)ethylene, *i.e*. 1:2, 1:1 and 2:1, only the binuclear compound {Cd[S_2_CN(*i*-Pr)CH_2_CH_2_OH)]_2_}_2_[1,2-bis­(4-pyrid­yl)ethylene]_3_, featuring one bridging and two terminally bound 1,2-bis­(4-pyrid­yl)ethylene ligands, could be isolated (Jotani *et al.*, 2016 ▸). Finally, in an unprecedented result, the original binuclear {Cd[S_2_CN(*i*-Pr)CH_2_CH_2_OH]_2_}_2_ aggregate was retained in the structure of [{Cd[S_2_CN(*i*-Pr)CH_2_CH_2_OH]_2_}_2_(3-pyridine­aldazine)]_2_ with two terminally bound 3-pyridine­aldazine ligands (Arman *et al.*, 2016 ▸). This is unusual as there are no precedents of adduct formation by the zinc-triad di­thio­carbamates that resulted in the retention of the original binuclear core (Tiekink, 2003 ▸).
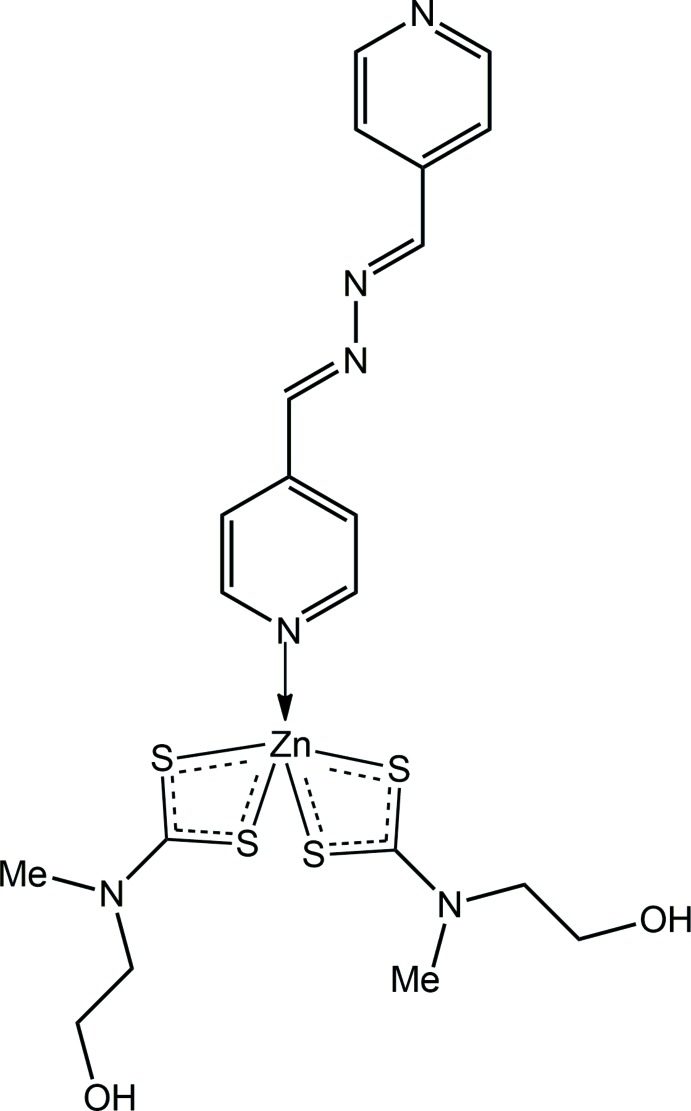


By contrast to the chemistry described above for cadmium di­thio­carbamates, no polymeric structures have been observed for zinc analogues with potentially bridging bipyridyl mol­ecules. Instead, only binuclear compounds of the general formula [Zn(S_2_CN*RR*′)_2_]_2_(*NN*), *i.e. R* = CH_2_CH_2_OH and *R*′ = Me, Et or CH_2_CH_2_OH for *NN* = 4,4′-bipyridyl (Benson *et al.*, 2007 ▸), *R* = *R*′ = CH_2_CH_2_OH and *NN* = pyrazine (Jotani *et al.*, 2017 ▸), and *R* = CH_2_CH_2_OH and *R*′ = Me for *NN* = (3-pyrid­yl)CH_2_N(H)C(=*Y*)C(=*Y*)N(H)CH_2_(3-pyrid­yl) where *Y* = O (Poplaukhin & Tiekink, 2010 ▸) and *Y* = S (Poplaukhin *et al.*, 2012 ▸). There are also several all-alkyl species adopting the binuclear motif with a notable example being the product of the reaction of [Zn(S_2_CN*R*_2_)_2_]_2_ with an excess of 1,2-bis­(4-pyrid­yl)ethyl­ene in which the binuclear species co-crystallized with an uncoordinated mol­ecule of 1,2-bis­(4-pyrid­yl)ethyl­ene (Lai & Tiekink, 2003 ▸). This difference in behaviour, *i.e*. polymer formation for cadmium but not for zinc di­thio­carbamates, is explained in terms of the larger size of cadmium *versus* zinc, which enables cadmium to increase its coordination number. In continuation of our studies in this area, the title compound, Zn[S_2_CN(Me)CH_2_CH_2_OH)]_2_(4-pyridine­alda­zine), (I), was isolated and shown to feature a terminally bound 4-pyridine­aldazine ligand. Herein, its crystal and mol­ecular structures are described as is an analysis of the calculated Hirshfeld surface.

## Structural commentary   

**Figure 1:**
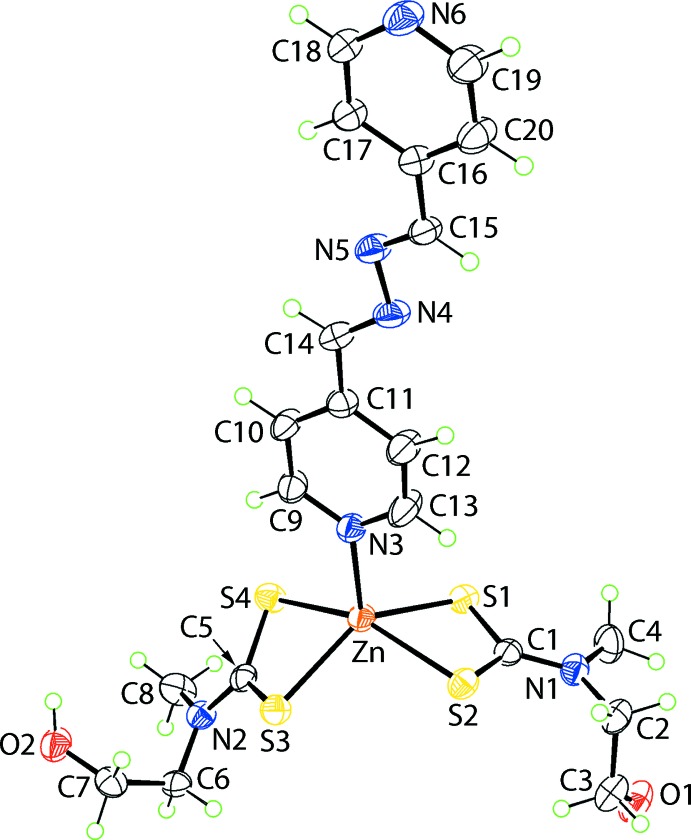
The mol­ecular structure of (I), showing the atom-labelling scheme and displacement ellipsoids at the 50% probability level.

The mol­ecular structure of (I) is shown in Fig. 1 ▸ and selected geometric parameters are given in Table 1 ▸. The zinc(II) atom is coordinated by two chelating di­thio­carbamate ligands and a nitro­gen atom derived from a monodentate 4-pyridine­aldazine ligand. There are relatively small differences in the Zn—S bond lengths formed by each di­thio­carbamate ligand, *i.e*. ΔZn—S = (Zn—S_long_ − Zn—S_short_) = 0.10 Å for the S1-di­thio­carbamate ligand which increases to *ca* 0.12 Å for the second ligand. This symmetric mode of coordination is reflected in the equivalence of the associated C—S bond lengths. The resulting NS_4_ donor set is highly distorted as shown by the value of τ of 0.32 which is inter­mediate between ideal square-pyramidal (τ = 0.0) and trigonal-bipyramidal (τ = 1.0) geometries (Addison *et al.*, 1984 ▸) but, with a tendency towards the former. In the square-pyramidal description, the zinc(II) centre lies 0.7107 (7) Å out of the plane defined by the four sulfur atoms [r.m.s. deviation = 0.1790 Å] in the direction of the pyridyl-N atom. The dihedral angle between the best plane through the four sulfur atoms and the coordinating pyridyl residue is 84.82 (9)°, consistent with a nearly symmetric perpendicular relationship. The 4-pyridine­aldazine mol­ecule has an all-*trans* conformation and is essentially planar as seen in the dihedral angle of 2.7 (3)° formed between the rings.

## Supra­molecular features   

**Figure 2:**
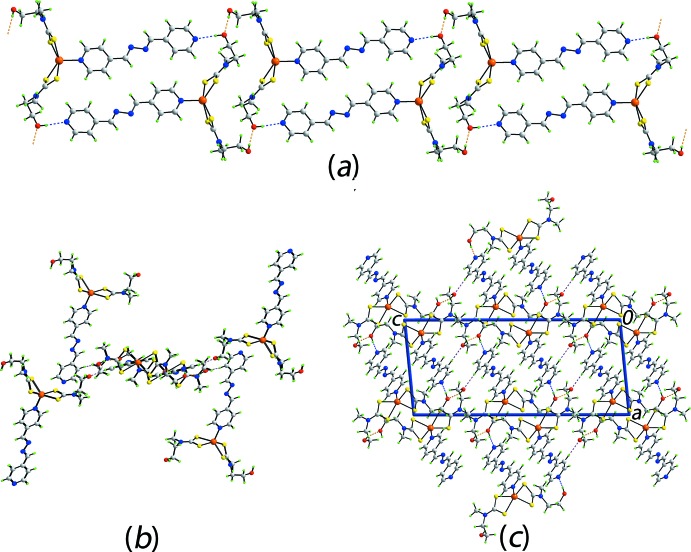
Mol­ecular packing for (I): (*a*) the supra­molecular double chain sustained by O—H⋯O and O—H⋯N hydrogen-bonding, shown as orange and blue, dashed lines, respectively, (*b*) a view of the immediate environment of one chain down the direction of propagation highlighting the role of C—H⋯O inter­actions (purple dashed lines) in sustaining the three-dimensional architecture and (*c*) a view of the unit-cell contents in projection down the *b* axis.

Both conventional and non-conventional hydrogen-bonding inter­actions feature in the crystal of (I), Table 2 ▸. Hydroxyl-O—H⋯O(hydrox­yl) hydrogen-bonds between centrosymmetrically related mol­ecules lead to 28-membered {⋯HOC_2_NCSZnSCNC_2_O}_2_ synthons. On either side of this aggregate are hydroxyl-O—H⋯N(pyrid­yl) hydrogen bonds leading to centrosymmetric 40-membered {⋯HOC_2_NCSZnNC_4_N_2_C_4_N}_2_ synthons. The result is a supra­molecular double-chain with the appearance of a ladder that extends along [1
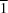
0], Fig. 2 ▸ *a*. Within the chains there are notable methylene-C—H⋯π(chelate ring) inter­actions, Table 2 ▸, which are garnering greater attention in the chemical crystallographic community (Tiekink, 2017 ▸). While the hydroxyl-O2 atom participates in acceptor O—H⋯O and donor O—H⋯N hydrogen-bonds, the O1 atom only forms a O—H⋯O hydrogen-bond. This being stated, this atom accepts a close pyridyl-C—H inter­action so that each chain is associated with four other chains. As seen from Fig. 2 ▸ *b*, the surrounding chains are inclined by approximately 90° and have orientations orthogonal to the reference chain. In this manner, a three-dimensional architecture is constructed as illustrated in Fig. 2 ▸ *c*.

## Hirshfeld surface analysis   

**Figure 3:**
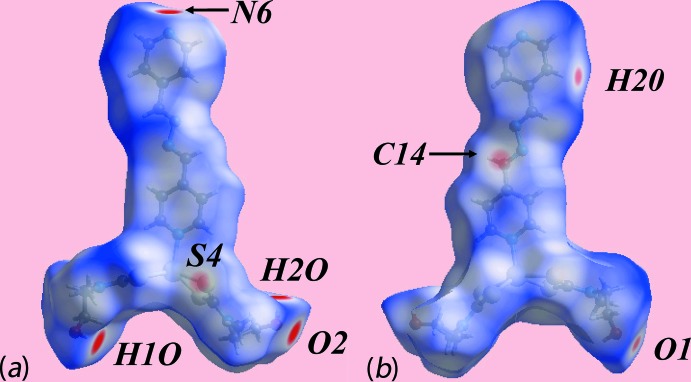
Two views of the Hirshfeld surface for (I) mapped over *d*_norm_ in the range −0.400 to 1.552 au.

**Figure 4:**
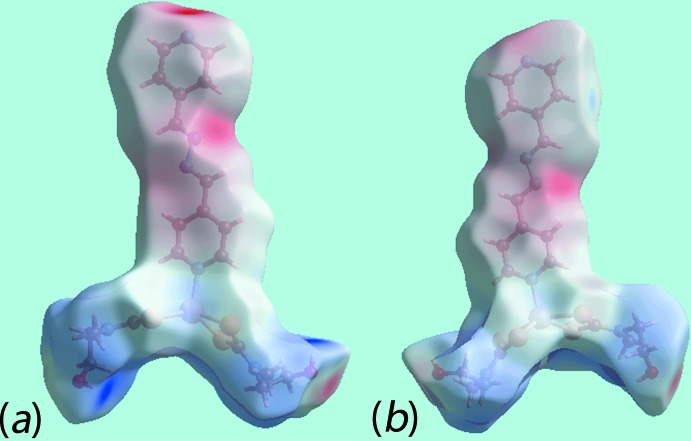
Two views of the Hirshfeld surface for (I) mapped over the electrostatic potential in the range ±0.151 au. The red and blue regions represent negative and positive electrostatic potentials, respectively.

**Figure 5:**
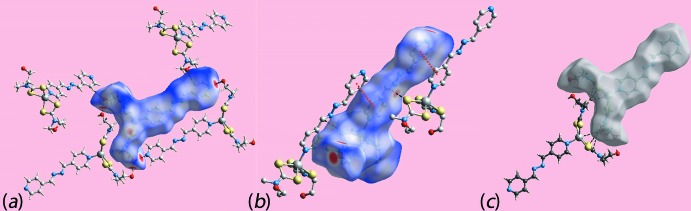
Views of Hirshfeld surface mapped over *d*_norm_ about a reference mol­ecule showing (*a*) inter­molecular O—H⋯O, O—H⋯N and C—H⋯O inter­actions as black dashed lines, (*b*) short inter-atomic S⋯C/C⋯S contacts and π—π stacking inter­actions as black and red lines, respectively (H atoms are omitted) and (*c*) C—H⋯π(chelate) inter­actions through short inter-atomic contacts involving the methylene-H6*B* atom with the Zn, S1 and C1 atoms of the chelate ring as black dashed lines.

Additional insight into the inter­molecular inter­actions influential in the crystal of (I) was obtained from an analysis of the Hirshfeld surfaces which were calculated in accord with a recent publication on related zinc di­thio­carbamate compounds (Jotani *et al.*, 2017 ▸). On the Hirshfeld surface mapped over *d*_norm_, Fig. 3 ▸, the donors and acceptors of the O—H⋯O and O—H⋯N hydrogen-bonds are viewed as bright-red spots near hydroxyl-H1*O*, H2*O*, hydroxyl-O2 and pyridyl-N6 atoms, located largely at the extremes of the mol­ecule. The presence of bright-red spots near the H1*O* and H2*O* atoms in Fig. 3 ▸ are also indicative of short inter-atomic H⋯H and C⋯H/H⋯C contacts, see Table 3 ▸. The diminutive-red spots near the methyl-C14, sulfur-S4, pyridyl-H20 and hydroxyl-O1 atoms characterize the influence of short inter-atomic C⋯S/S⋯C contacts, Table 3 ▸, and inter­molecular pyridine-C20—H20⋯O1 inter­actions. The donors and acceptors of the above inter­molecular inter­actions are also represented with blue and red regions on the Hirshfeld surface mapped over electrostatic potential shown in Fig. 4 ▸. The immediate environments about a reference mol­ecule within *d*_norm_-mapped Hirshfeld surface highlighting inter­molecular O—H⋯O, O—H⋯N and C—H⋯O, short inter-atomic C⋯S/S⋯C contacts, π—π stacking inter­actions and C—H⋯π(chelate) inter­actions are illus­trated in Fig. 5 ▸ *a*–*c*, respectively.

**Figure 6:**
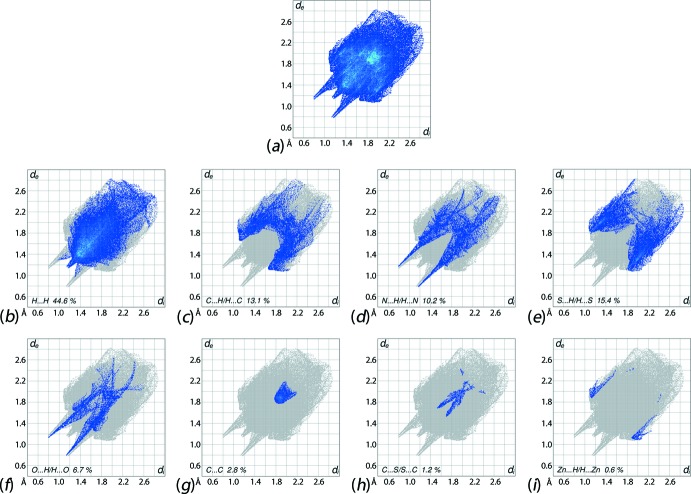
The full two-dimensional fingerprint plot for (I) and fingerprint plots delineated into (*b*) H⋯H, (*c*) C⋯H/H⋯C, (*d*) N⋯H/H⋯N, (*e*) S⋯H/H⋯S, (*f*) O⋯H/H⋯O, (*g*) C⋯C, (*h*) C⋯S/S⋯C and (*i*) Zn⋯H/H⋯Zn contacts.

**Figure 7:**
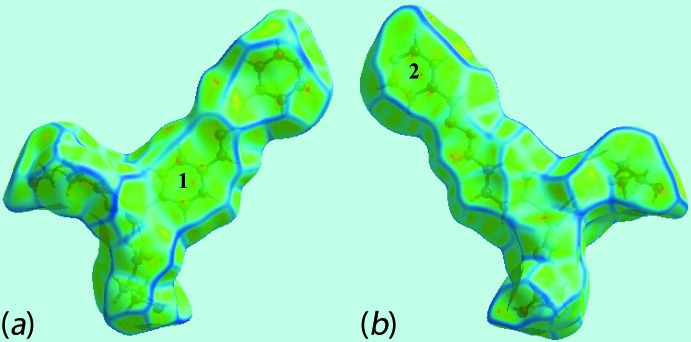
Two views of Hirshfeld surface mapped over curvedness showing flat regions over pyridyl-(N3,C9–C13) and (N6, C15–C20) rings with labels **1** and **2**, respectively.

The overall two dimensional fingerprint plot, Fig. 6 ▸ *a*, and those delineated into H⋯H, C⋯H/H⋯C, N⋯H/H⋯N, S⋯H/H⋯S, O⋯H/H⋯O, C⋯C, C⋯S/S⋯C and Zn⋯H/H⋯Zn contacts (McKinnon *et al.*, 2007 ▸) are illustrated in Fig. 6 ▸ *b*–*i*, respectively; the relative contributions from different inter-atomic contacts to the Hirshfeld surfaces are summarized in Table 4 ▸. The pair of adjacent short spikes at *d*_e_ + *d*_i_ ∼ 2.2 Å flanked by the broad spikes with tips at *d*_e_ + *d*_i_ ∼ 2.3 Å in the fingerprint plot delineated into H⋯H contacts are due to short inter-atomic H⋯H contacts, Fig. 6 ▸ *b.* The forceps-like tips at *d*_e_ + *d*_i_ ∼ 2.8 Å in the fingerprint plot delineated into C⋯H/H⋯C contacts, Fig. 6 ▸ *c*, are due to the presence of some short inter-atomic contacts involving these atoms, Table 3 ▸. The effect of the inter­molecular C—H⋯π(chelate) inter­actions is also reflected by the short inter-atomic contacts formed by the methylene-C6 with the Zn atom, and methylene-H6*B* with the Zn, S1 and C1 atoms of the chelate ring, Fig. 6 ▸ *c*, 6*e*, 6*i*, and Table 2 ▸. The two pairs of adjacent long spikes on the fingerprint plots delineated into N⋯H/H⋯N and O⋯H/H⋯O contacts, Fig. 6 ▸ *d* and 6*f*, with the pair of tips at *d*_e_ + *d*_i_ ∼ 2.0 Å and *d*_e_ + *d*_i_ ∼ 1.9 Å, respectively, indicate the presence of conventional O—H⋯O and O—H⋯N hydrogen-bonds in the structure. The points corresponding to short inter-atomic N⋯H/H⋯N contacts, Table 3 ▸, are merged within the plot in Fig. 6 ▸ *d*. The pattern of aligned green points superimposed on the forceps-like distribution of blue points in the S⋯H/H⋯S delineated fingerprint plot in Fig. 6 ▸ *e* characterize the presence of short inter-atomic S⋯H/H⋯S contacts, Table 3 ▸, and C—H⋯π (chelate) inter­actions, Fig. 5 ▸ *c*. The C—H⋯O inter­actions appear as the distribution of points in the short parabolic form attached to each of the spikes on the outer side of fingerprint plot delineated into O⋯H/H⋯O contacts, Fig. 6 ▸ *f*, with (*d*_e_ + *d*_i_)_min_ ∼ 2.3 Å. The parabolic distribution of points in the (*d*_e_ = *d*_i_) ∼ 1.8–2.0 Å range in the fingerprint plot delineated into C⋯C contacts, Fig. 6 ▸ *g*, indicate the existence of weak π–π stacking inter­actions between the pyridyl-(N3,C9–C13) and (N6, C15–C20)^i^ rings [*Cg*⋯*Cg* ^i^ = 3.901 (3) Å; symmetry code: (i) = *x*, 1 + *y*, *z*]. This observation is also viewed as the flat region around these rings in the Hirshfeld surfaces mapped over curvedness in Fig. 7 ▸. Both the C⋯S/S⋯C and Zn⋯H/H⋯Zn contacts make small but discernible contributions of 1.2 and 0.6% to the Hirshfeld surface, respectively, which are manifested as the pair of the short spikes in the centre of Fig. 6 ▸ *h*, with their tips at *d*_e_ + *d*_i_ ∼ 3.2 Å, and wings in Fig. 6 ▸ *i*. The low contribution from other contacts summarized in Table 4 ▸ have no significant influence on the mol­ecular packing owing to their long separations.

## Database survey   

A search of the Cambridge Structural Database (Version 5.38, May 2017 update; Groom *et al.*, 2016 ▸) showed there were over 145 examples of metal complexes/main-group element compounds containing the 4-pyridine­aldazine mol­ecule. Bridging modes were observed in both cadmium(II) (Lai & Tiekink, 2006 ▸) and nickel(II) (*e.g*. Berdugo & Tiekink, 2009 ▸) di­thio­phosphate [^−^S_2_P(O*R*)_2_] derivatives, indicating bridging modes are possible in the presence of 1,1-di­thiol­ate co-ligands. There were six examples of structures where 4-pyridine­aldazine was present in the crystal but was non-coordinating, and two where the ligand was terminally bound as in (I), *i.e*. the cadmium analogue of (I) and in a structure particularly worth highlighting as both a terminally bound ligand as well as a non-coordinating mol­ecule of 4-pyridine­aldazine are present, namely [Zn(OH_2_)_2_[O(H)Me]_2_(4-pyridine­aldazine)_2_](ClO_4_)_2_·4-pyrid­ine­aldazine, 1.72MeOH, 1.28H_2_O (Shoshnik *et al.*, 2005 ▸). In summary, the 4-pyridine­aldazine mol­ecule is usually found to be bridging, a conclusion vindicated by this mode of coord­in­ation being observed in about 95% of structures having 4-pyridine­aldazine. While one might be tempted to ascribe the unusual behaviour of 4-pyridine­aldazine in (I) and the cadmium(II) analogue to the influence of hydrogen-bonding associated with the di­thio­carbamate ligand, it is salutatory to recall that the sole example of a monodentate bipyridyl ligand is found in the structure of Zn[S_2_CN(*n*-Pr)_2_]_2_(4,4′-bipyrid­yl) (Klevtsova *et al.*, 2001 ▸), where there is no possibility of conventional hydrogen-bonding inter­actions; the binuclear species, {Zn[S_2_CN(*n*-Pr)_2_]_2_}_2_(4,4′-bipyrid­yl), was characterized in the same study.

## Synthesis and crystallization   

Compound (I) was prepared following the standard literature procedure whereby the 1:1 reaction of Zn[S_2_CN(Me)CH_2_CH_2_OH]_2_ (Howie *et al.*, 2008 ▸) and 4-pyridine­aldazine (Sigma Aldrich). Yellow crystals of (I) were obtained from the slow evaporation of a chloro­form/aceto­nitrile (3/1) solution.

## Refinement details   

Crystal data, data collection and structure refinement details are summarized in Table 5 ▸. The carbon-bound H atoms were placed in calculated positions (C—H = 0.95–0.99 Å) and were included in the refinement in the riding-model approximation, with *U*_iso_(H) set to 1.2–1.5*U*_eq_(C). The O-bound H atoms were located in a difference-Fourier map but were refined with distance restraint of O—H = 0.84±0.01 Å, and with *U*_iso_(H) set to 1.5*U*_eq_(O).

## Supplementary Material

Crystal structure: contains datablock(s) I, global. DOI: 10.1107/S2056989017012725/hb7702sup1.cif

Structure factors: contains datablock(s) I. DOI: 10.1107/S2056989017012725/hb7702Isup2.hkl

CCDC reference: 1572824

Additional supporting information:  crystallographic information; 3D view; checkCIF report

## Figures and Tables

**Table 1:** Selected geometric parameters (Å, °)

Zn—S1	2.4152 (12)	Zn—S4	2.5162 (11)
Zn—S2	2.5152 (11)	Zn—N3	2.068 (3)
Zn—S3	2.3890 (12)		
			
S1—Zn—S3	136.48 (4)	S2—Zn—S4	155.56 (4)

**Table 2:** Hydrogen-bond geometry (Å, °) *Cg*1 is the centroid of the Zn/S1/S2/C1 ring.

*D*—H⋯*A*	*D*—H	H⋯*A*	*D*⋯*A*	*D*—H⋯*A*
O1—H1*O*⋯O2^i^	0.85 (5)	1.92 (5)	2.721 (5)	158 (5)
O2—H2*O*⋯N6^ii^	0.84 (4)	1.95 (4)	2.769 (5)	163 (5)
C20—H20⋯O1^iii^	0.95	2.32	3.233 (6)	162
C6—H6*B*⋯*Cg*1^i^	0.99	2.59	3.540 (4)	162

Symmetry codes: (i) 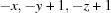; (ii) 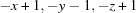; (iii) 
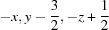
.

**Table 3:** Summary of short inter-atomic contacts (Å) in (I)

Contact	Distance	Symmetry operation
H1*O*⋯H2*O*	2.21 (7)	−*x*, 1 − *y*, 1 − *z*
H4*B*⋯H13	2.30	−*x*, 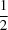 + *y*, 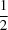 − *z*
Zn⋯C6	3.835 (4)	−*x*, 1 − *y*, 1 − *z*
Zn⋯H6*B*	3.00	−*x*, 1 − *y*, 1 − *z*
C1⋯H6*B*	2.88	−*x*, 1 − *y*, 1 − *z*
S1⋯H6*B*	2.92	−*x*, 1 − *y*, 1 − *z*
S1⋯H15	2.98	*x*, 1 + *y*, *z*
S2⋯H7*B*	2.89	−*x*, −*y*, 1 − *z*
S4⋯C14	3.217 (4)	*x*, 1 + *y*, *z*
C2⋯H4*A*	2.88	−*x*, − 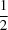 + *y*, 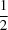 − *z*
C5⋯H18	2.77	1 − *x*, 1 − *y*, 1 − *z*
C18⋯H2*O*	2.89 (5)	1 − *x*, 1 − *y*, 1 − *z*
C19⋯H2*O*	2.85 (4)	1 − *x*, 1 − *y*, 1 − *z*
N5⋯H8*A*	2.73	1 − *x*, −*y*, 1 − *z*

**Table 4:** Percentage contributions of inter-atomic contacts to the Hirshfeld surfaces for (I)

Contact	Percentage contribution
H⋯H	44.6
S⋯H/H⋯S	15.4
C⋯H/H⋯C	13.1
N⋯H/H⋯N	10.2
O⋯H/H⋯O	6.7
C⋯C	2.8
S⋯N/N⋯S	2.8
S⋯S	1.5
C⋯S/S⋯C	1.2
C⋯N/N⋯C	1.0
Zn⋯H/H⋯Zn	0.6
Zn⋯S/S⋯Zn	0.1

**Table 5:** Experimental details

Crystal data	
Chemical formula	[Zn(C_4_H_8_NOS_2_)_2_C_12_H_10_N_4_)]
*M*_r_	576.08
Crystal system, space group	Monoclinic, *P*2_1_/*c*
Temperature (K)	153
*a*, *b*, *c* (Å)	11.499 (4), 8.5710 (19), 25.945 (7)
β (°)	95.515 (8)
*V* (Å^3^)	2545.3 (13)
*Z*	4
Radiation type	Mo *K*α
μ (mm^−1^)	1.32
Crystal size (mm)	0.40 × 0.18 × 0.15

Data collection	
Diffractometer	Rigaku AFC12K/SATURN724
Absorption correction	Multi-scan (*ABSCOR*; Higashi, 1995 ▸)
*T*_min_, *T*_max_	0.575, 1
No. of measured, independent and observed [*I* > 2σ(*I*)] reflections	25373, 4485, 4180
*R*_int_	0.044
(sin θ/λ)_max_ (Å^−1^)	0.595

Refinement	
*R*[*F* ^2^ > 2σ(*F* ^2^)], *wR*(*F* ^2^), *S*	0.050, 0.132, 1.13
No. of reflections	4485
No. of parameters	306
No. of restraints	2
H-atom treatment	H atoms treated by a mixture of independent and constrained refinement
Δρ_max_, Δρ_min_ (e Å^−3^)	0.72, −0.44

Computer programs: *CrystalClear* (Molecular Structure Corporation & Rigaku, 2005 ▸), *SHELXS* (Sheldrick, 2008 ▸), *SHELXL2014/7* (Sheldrick, 2015 ▸), *ORTEP-3 for Windows* (Farrugia, 2012 ▸) and *DIAMOND* (Brandenburg, 2006 ▸), *publCIF* (Westrip, 2010 ▸).

## supplementary crystallographic information

### Crystal data

**Table:**

[Zn(C_4_H_8_NOS_2_)_2_C_12_H_10_N_4_)]	*F*(000) = 1192
*M**_r_* = 576.08	*D*_x_ = 1.503 Mg m^−^^3^
Monoclinic, *P*2_1_/*c*	Mo *K*α radiation, λ = 0.71069 Å
*a* = 11.499 (4) Å	Cell parameters from 1535 reflections
*b* = 8.5710 (19) Å	θ = 3.1–30.3°
*c* = 25.945 (7) Å	µ = 1.32 mm^−^^1^
β = 95.515 (8)°	*T* = 153 K
*V* = 2545.3 (13) Å^3^	Prism, yellow
*Z* = 4	0.40 × 0.18 × 0.15 mm

### Data collection

**Table:**

Rigaku AFC12K/SATURN724 diffractometer	4485 independent reflections
Radiation source: fine-focus sealed tube	4180 reflections with *I* > 2σ(*I*)
Graphite monochromator	*R*_int_ = 0.044
ω scans	θ_max_ = 25.0°, θ_min_ = 2.3°
Absorption correction: multi-scan (ABSCOR; Higashi, 1995)	*h* = −13→13
*T*_min_ = 0.575, *T*_max_ = 1	*k* = −10→8
25373 measured reflections	*l* = −30→30

### Refinement

**Table:**

Refinement on *F*^2^	2 restraints
Least-squares matrix: full	Hydrogen site location: mixed
*R*[*F*^2^ > 2σ(*F*^2^)] = 0.050	H atoms treated by a mixture of independent and constrained refinement
*wR*(*F*^2^) = 0.132	*w* = 1/[σ^2^(*F*_o_^2^) + (0.0663*P*)^2^ + 3.198*P*] where *P* = (*F*_o_^2^ + 2*F*_c_^2^)/3
*S* = 1.13	(Δ/σ)_max_ = 0.001
4485 reflections	Δρ_max_ = 0.72 e Å^−^^3^
306 parameters	Δρ_min_ = −0.44 e Å^−^^3^

### Special details

**Table:**

Geometry. All esds (except the esd in the dihedral angle between two l.s. planes) are estimated using the full covariance matrix. The cell esds are taken into account individually in the estimation of esds in distances, angles and torsion angles; correlations between esds in cell parameters are only used when they are defined by crystal symmetry. An approximate (isotropic) treatment of cell esds is used for estimating esds involving l.s. planes.

### Fractional atomic coordinates and isotropic or equivalent isotropic displacement parameters (Å^2^)

**Table:**

	*x*	*y*	*z*	*U*_iso_*/*U*_eq_	
Zn	0.13806 (4)	0.15577 (5)	0.42882 (2)	0.03058 (16)	
S1	0.13958 (8)	0.27822 (11)	0.34505 (4)	0.0337 (2)	
S2	−0.04566 (8)	0.07162 (11)	0.37564 (4)	0.0327 (2)	
S3	0.04838 (8)	0.20445 (11)	0.50658 (4)	0.0332 (2)	
S4	0.26964 (8)	0.34274 (11)	0.48156 (4)	0.0349 (2)	
O1	−0.2678 (3)	0.3986 (4)	0.25882 (13)	0.0546 (8)	
H1O	−0.225 (4)	0.462 (5)	0.277 (2)	0.082*	
O2	0.1675 (3)	0.4188 (3)	0.66339 (11)	0.0447 (7)	
H2O	0.230 (3)	0.373 (6)	0.658 (2)	0.067*	
N1	−0.0546 (3)	0.2180 (4)	0.28469 (12)	0.0346 (7)	
N2	0.1419 (3)	0.4612 (3)	0.55174 (11)	0.0305 (7)	
N3	0.2411 (3)	−0.0416 (4)	0.42704 (12)	0.0325 (7)	
N4	0.4206 (3)	−0.5525 (4)	0.38299 (13)	0.0377 (8)	
N5	0.4931 (3)	−0.6843 (4)	0.38951 (13)	0.0363 (7)	
N6	0.6562 (3)	−1.2152 (4)	0.35773 (14)	0.0437 (8)	
C1	0.0057 (3)	0.1899 (4)	0.33010 (14)	0.0291 (8)	
C2	−0.1697 (3)	0.1479 (5)	0.27017 (16)	0.0396 (9)	
H2A	−0.1744	0.0463	0.2880	0.048*	
H2B	−0.1786	0.1282	0.2324	0.048*	
C3	−0.2681 (4)	0.2508 (5)	0.28404 (17)	0.0472 (10)	
H3A	−0.3435	0.1979	0.2741	0.057*	
H3B	−0.2612	0.2670	0.3220	0.057*	
C4	−0.0090 (4)	0.3239 (6)	0.24694 (16)	0.0473 (11)	
H4A	0.0029	0.4277	0.2624	0.071*	
H4B	−0.0650	0.3308	0.2161	0.071*	
H4C	0.0656	0.2837	0.2372	0.071*	
C5	0.1526 (3)	0.3476 (4)	0.51757 (14)	0.0293 (8)	
C6	0.0441 (3)	0.4690 (4)	0.58335 (14)	0.0329 (8)	
H6A	−0.0271	0.4303	0.5628	0.040*	
H6B	0.0304	0.5793	0.5924	0.040*	
C7	0.0636 (3)	0.3757 (4)	0.63208 (14)	0.0348 (8)	
H7A	−0.0042	0.3895	0.6524	0.042*	
H7B	0.0682	0.2638	0.6230	0.042*	
C8	0.2286 (4)	0.5857 (5)	0.55967 (17)	0.0424 (10)	
H8A	0.2938	0.5502	0.5840	0.064*	
H8B	0.1926	0.6778	0.5739	0.064*	
H8C	0.2577	0.6125	0.5265	0.064*	
C9	0.3348 (3)	−0.0675 (4)	0.46063 (15)	0.0366 (9)	
H9	0.3551	0.0079	0.4868	0.044*	
C10	0.4029 (3)	−0.1992 (5)	0.45873 (15)	0.0364 (9)	
H10	0.4690	−0.2131	0.4832	0.044*	
C11	0.3752 (3)	−0.3110 (4)	0.42118 (14)	0.0320 (8)	
C12	0.2771 (4)	−0.2850 (6)	0.38681 (19)	0.0553 (13)	
H12	0.2538	−0.3591	0.3606	0.066*	
C13	0.2146 (4)	−0.1507 (5)	0.39140 (19)	0.0557 (13)	
H13	0.1477	−0.1343	0.3675	0.067*	
C14	0.4440 (3)	−0.4535 (4)	0.41878 (15)	0.0341 (8)	
H14	0.5072	−0.4724	0.4444	0.041*	
C15	0.4595 (3)	−0.7935 (4)	0.35868 (15)	0.0328 (8)	
H15	0.3919	−0.7803	0.3350	0.039*	
C16	0.5253 (3)	−0.9406 (4)	0.35960 (14)	0.0315 (8)	
C17	0.6222 (3)	−0.9662 (4)	0.39506 (15)	0.0329 (8)	
H17	0.6453	−0.8902	0.4207	0.039*	
C18	0.6836 (3)	−1.1017 (4)	0.39256 (15)	0.0349 (8)	
H18	0.7498	−1.1169	0.4169	0.042*	
C19	0.5620 (4)	−1.1910 (5)	0.32486 (18)	0.0481 (11)	
H19	0.5398	−1.2707	0.3004	0.058*	
C20	0.4943 (4)	−1.0576 (5)	0.32386 (18)	0.0447 (10)	
H20	0.4281	−1.0462	0.2993	0.054*	

### Atomic displacement parameters (Å^2^)

**Table:**

	*U*^11^	*U*^22^	*U*^33^	*U*^12^	*U*^13^	*U*^23^
Zn	0.0318 (3)	0.0277 (3)	0.0314 (3)	0.00704 (16)	−0.00087 (18)	−0.00366 (16)
S1	0.0302 (5)	0.0343 (5)	0.0365 (5)	0.0010 (4)	0.0022 (4)	−0.0003 (4)
S2	0.0322 (5)	0.0286 (5)	0.0366 (5)	0.0015 (4)	−0.0014 (4)	0.0018 (4)
S3	0.0317 (5)	0.0317 (5)	0.0360 (5)	−0.0020 (4)	0.0025 (4)	−0.0042 (4)
S4	0.0351 (5)	0.0321 (5)	0.0381 (5)	−0.0023 (4)	0.0063 (4)	−0.0061 (4)
O1	0.055 (2)	0.0464 (18)	0.0575 (19)	0.0100 (15)	−0.0213 (15)	0.0022 (15)
O2	0.0492 (17)	0.0422 (17)	0.0404 (15)	0.0154 (13)	−0.0074 (13)	−0.0085 (13)
N1	0.0365 (17)	0.0351 (17)	0.0314 (16)	0.0016 (14)	−0.0011 (13)	0.0012 (13)
N2	0.0322 (16)	0.0243 (15)	0.0347 (16)	0.0031 (12)	0.0012 (13)	−0.0011 (13)
N3	0.0324 (16)	0.0309 (16)	0.0332 (16)	0.0054 (13)	−0.0014 (13)	−0.0044 (13)
N4	0.0312 (17)	0.0334 (18)	0.0484 (19)	0.0091 (14)	0.0029 (14)	−0.0042 (15)
N5	0.0290 (16)	0.0309 (17)	0.0480 (19)	0.0063 (13)	−0.0007 (14)	−0.0040 (15)
N6	0.0402 (19)	0.0330 (18)	0.056 (2)	0.0101 (15)	−0.0052 (16)	−0.0051 (16)
C1	0.0294 (18)	0.0252 (17)	0.0321 (19)	0.0043 (14)	0.0007 (15)	−0.0046 (15)
C2	0.036 (2)	0.038 (2)	0.042 (2)	−0.0015 (17)	−0.0091 (18)	−0.0033 (17)
C3	0.037 (2)	0.053 (3)	0.049 (2)	−0.0003 (19)	−0.0100 (19)	0.003 (2)
C4	0.055 (3)	0.055 (3)	0.031 (2)	−0.002 (2)	0.0009 (19)	0.0096 (19)
C5	0.0303 (19)	0.0264 (18)	0.0300 (18)	0.0070 (14)	−0.0034 (15)	0.0004 (14)
C6	0.0304 (18)	0.0319 (19)	0.0366 (19)	0.0075 (15)	0.0035 (15)	−0.0026 (16)
C7	0.043 (2)	0.0253 (18)	0.037 (2)	0.0048 (16)	0.0075 (17)	−0.0026 (16)
C8	0.042 (2)	0.033 (2)	0.054 (2)	−0.0107 (17)	0.0106 (19)	−0.0143 (19)
C9	0.040 (2)	0.033 (2)	0.035 (2)	0.0047 (17)	−0.0052 (17)	−0.0039 (16)
C10	0.033 (2)	0.033 (2)	0.041 (2)	0.0076 (16)	−0.0083 (17)	0.0012 (17)
C11	0.0304 (19)	0.0296 (19)	0.0359 (19)	0.0018 (15)	0.0028 (16)	−0.0006 (16)
C12	0.049 (3)	0.051 (3)	0.060 (3)	0.022 (2)	−0.022 (2)	−0.030 (2)
C13	0.052 (3)	0.049 (3)	0.060 (3)	0.025 (2)	−0.027 (2)	−0.021 (2)
C14	0.0252 (18)	0.033 (2)	0.044 (2)	0.0046 (15)	0.0017 (16)	0.0054 (17)
C15	0.0259 (18)	0.034 (2)	0.038 (2)	0.0058 (15)	0.0007 (15)	0.0005 (17)
C16	0.0267 (18)	0.0282 (19)	0.040 (2)	0.0012 (15)	0.0032 (15)	−0.0001 (16)
C17	0.0304 (19)	0.0283 (19)	0.039 (2)	−0.0001 (15)	0.0001 (16)	−0.0018 (16)
C18	0.0301 (19)	0.034 (2)	0.040 (2)	0.0022 (16)	−0.0017 (16)	0.0025 (17)
C19	0.046 (2)	0.038 (2)	0.058 (3)	0.0056 (19)	−0.007 (2)	−0.013 (2)
C20	0.034 (2)	0.038 (2)	0.059 (3)	0.0025 (17)	−0.0106 (19)	−0.010 (2)

### Geometric parameters (Å, º)

**Table:**

Zn—S1	2.4152 (12)	C4—H4A	0.9800
Zn—S2	2.5152 (11)	C4—H4B	0.9800
Zn—S3	2.3890 (12)	C4—H4C	0.9800
Zn—S4	2.5162 (11)	C6—C7	1.495 (5)
Zn—N3	2.068 (3)	C6—H6A	0.9900
S1—C1	1.726 (4)	C6—H6B	0.9900
S2—C1	1.705 (4)	C7—H7A	0.9900
S3—C5	1.720 (4)	C7—H7B	0.9900
S4—C5	1.711 (4)	C8—H8A	0.9800
O1—C3	1.426 (5)	C8—H8B	0.9800
O1—H1O	0.841 (10)	C8—H8C	0.9800
O2—C7	1.428 (5)	C9—C10	1.378 (5)
O2—H2O	0.838 (10)	C9—H9	0.9500
N1—C1	1.331 (5)	C10—C11	1.382 (5)
N1—C4	1.468 (5)	C10—H10	0.9500
N1—C2	1.469 (5)	C11—C12	1.387 (5)
N2—C5	1.331 (5)	C11—C14	1.460 (5)
N2—C6	1.456 (5)	C12—C13	1.369 (6)
N2—C8	1.461 (5)	C12—H12	0.9500
N3—C13	1.330 (5)	C13—H13	0.9500
N3—C9	1.337 (5)	C14—H14	0.9500
N4—C14	1.267 (5)	C15—C16	1.470 (5)
N4—N5	1.405 (4)	C15—H15	0.9500
N5—C15	1.267 (5)	C16—C20	1.389 (5)
N6—C19	1.329 (5)	C16—C17	1.393 (5)
N6—C18	1.344 (5)	C17—C18	1.364 (5)
C2—C3	1.506 (6)	C17—H17	0.9500
C2—H2A	0.9900	C18—H18	0.9500
C2—H2B	0.9900	C19—C20	1.383 (6)
C3—H3A	0.9900	C19—H19	0.9500
C3—H3B	0.9900	C20—H20	0.9500
			
N3—Zn—S3	117.15 (9)	N2—C6—H6A	109.0
N3—Zn—S1	106.36 (9)	C7—C6—H6A	109.0
S1—Zn—S3	136.48 (4)	N2—C6—H6B	109.0
N3—Zn—S2	101.88 (9)	C7—C6—H6B	109.0
S3—Zn—S2	96.05 (4)	H6A—C6—H6B	107.8
S1—Zn—S2	73.10 (4)	O2—C7—C6	113.1 (3)
N3—Zn—S4	102.55 (9)	O2—C7—H7A	109.0
S3—Zn—S4	73.47 (4)	C6—C7—H7A	109.0
S1—Zn—S4	99.01 (4)	O2—C7—H7B	109.0
S2—Zn—S4	155.56 (4)	C6—C7—H7B	109.0
C1—S1—Zn	85.88 (13)	H7A—C7—H7B	107.8
C1—S2—Zn	83.17 (12)	N2—C8—H8A	109.5
C5—S3—Zn	85.07 (13)	N2—C8—H8B	109.5
C5—S4—Zn	81.33 (12)	H8A—C8—H8B	109.5
C3—O1—H1O	111 (4)	N2—C8—H8C	109.5
C7—O2—H2O	118 (4)	H8A—C8—H8C	109.5
C1—N1—C4	120.9 (3)	H8B—C8—H8C	109.5
C1—N1—C2	122.2 (3)	N3—C9—C10	122.5 (3)
C4—N1—C2	116.9 (3)	N3—C9—H9	118.7
C5—N2—C6	122.3 (3)	C10—C9—H9	118.7
C5—N2—C8	121.5 (3)	C9—C10—C11	120.0 (3)
C6—N2—C8	116.2 (3)	C9—C10—H10	120.0
C13—N3—C9	116.9 (3)	C11—C10—H10	120.0
C13—N3—Zn	119.8 (3)	C10—C11—C12	117.4 (3)
C9—N3—Zn	123.3 (2)	C10—C11—C14	121.4 (3)
C14—N4—N5	111.6 (3)	C12—C11—C14	121.2 (3)
C15—N5—N4	112.1 (3)	C13—C12—C11	118.8 (4)
C19—N6—C18	116.3 (3)	C13—C12—H12	120.6
N1—C1—S2	122.4 (3)	C11—C12—H12	120.6
N1—C1—S1	119.8 (3)	N3—C13—C12	124.4 (4)
S2—C1—S1	117.8 (2)	N3—C13—H13	117.8
N1—C2—C3	112.3 (3)	C12—C13—H13	117.8
N1—C2—H2A	109.2	N4—C14—C11	120.9 (3)
C3—C2—H2A	109.2	N4—C14—H14	119.5
N1—C2—H2B	109.2	C11—C14—H14	119.5
C3—C2—H2B	109.2	N5—C15—C16	119.9 (3)
H2A—C2—H2B	107.9	N5—C15—H15	120.1
O1—C3—C2	112.1 (4)	C16—C15—H15	120.1
O1—C3—H3A	109.2	C20—C16—C17	117.7 (3)
C2—C3—H3A	109.2	C20—C16—C15	120.7 (3)
O1—C3—H3B	109.2	C17—C16—C15	121.6 (3)
C2—C3—H3B	109.2	C18—C17—C16	119.2 (3)
H3A—C3—H3B	107.9	C18—C17—H17	120.4
N1—C4—H4A	109.5	C16—C17—H17	120.4
N1—C4—H4B	109.5	N6—C18—C17	124.0 (3)
H4A—C4—H4B	109.5	N6—C18—H18	118.0
N1—C4—H4C	109.5	C17—C18—H18	118.0
H4A—C4—H4C	109.5	N6—C19—C20	124.3 (4)
H4B—C4—H4C	109.5	N6—C19—H19	117.8
N2—C5—S4	120.7 (3)	C20—C19—H19	117.8
N2—C5—S3	121.7 (3)	C19—C20—C16	118.5 (4)
S4—C5—S3	117.7 (2)	C19—C20—H20	120.8
N2—C6—C7	113.0 (3)	C16—C20—H20	120.8
			
C14—N4—N5—C15	170.1 (4)	Zn—N3—C9—C10	−179.6 (3)
C4—N1—C1—S2	178.3 (3)	N3—C9—C10—C11	−0.2 (6)
C2—N1—C1—S2	−0.6 (5)	C9—C10—C11—C12	−0.7 (6)
C4—N1—C1—S1	−0.1 (5)	C9—C10—C11—C14	−178.6 (4)
C2—N1—C1—S1	−179.1 (3)	C10—C11—C12—C13	0.8 (7)
Zn—S2—C1—N1	−175.9 (3)	C14—C11—C12—C13	178.8 (5)
Zn—S2—C1—S1	2.55 (18)	C9—N3—C13—C12	−0.6 (8)
Zn—S1—C1—N1	175.9 (3)	Zn—N3—C13—C12	179.8 (4)
Zn—S1—C1—S2	−2.64 (19)	C11—C12—C13—N3	−0.2 (9)
C1—N1—C2—C3	92.4 (4)	N5—N4—C14—C11	−176.8 (3)
C4—N1—C2—C3	−86.6 (4)	C10—C11—C14—N4	−177.2 (4)
N1—C2—C3—O1	59.9 (4)	C12—C11—C14—N4	4.9 (6)
C6—N2—C5—S4	−179.6 (2)	N4—N5—C15—C16	179.5 (3)
C8—N2—C5—S4	0.3 (5)	N5—C15—C16—C20	−175.7 (4)
C6—N2—C5—S3	2.3 (5)	N5—C15—C16—C17	2.4 (6)
C8—N2—C5—S3	−177.8 (3)	C20—C16—C17—C18	1.4 (6)
Zn—S4—C5—N2	−163.6 (3)	C15—C16—C17—C18	−176.8 (3)
Zn—S4—C5—S3	14.54 (17)	C19—N6—C18—C17	−1.1 (6)
Zn—S3—C5—N2	162.9 (3)	C16—C17—C18—N6	−0.4 (6)
Zn—S3—C5—S4	−15.21 (18)	C18—N6—C19—C20	1.5 (7)
C5—N2—C6—C7	86.1 (4)	N6—C19—C20—C16	−0.5 (7)
C8—N2—C6—C7	−93.8 (4)	C17—C16—C20—C19	−1.0 (6)
N2—C6—C7—O2	56.2 (4)	C15—C16—C20—C19	177.2 (4)
C13—N3—C9—C10	0.8 (6)		

### Hydrogen-bond geometry (Å, º)

Cg1 is the centroid of the Zn/S1/S2/C1 ring.

**Table:**

*D*—H···*A*	*D*—H	H···*A*	*D*···*A*	*D*—H···*A*
O1—H1*O*···O2^i^	0.85 (5)	1.92 (5)	2.721 (5)	158 (5)
O2—H2*O*···N6^ii^	0.84 (4)	1.95 (4)	2.769 (5)	163 (5)
C20—H20···O1^iii^	0.95	2.32	3.233 (6)	162
C6—H6*B*···*Cg*1^i^	0.99	2.59	3.540 (4)	162

Symmetry codes: (i) −*x*, −*y*+1, −*z*+1; (ii) −*x*+1, −*y*−1, −*z*+1; (iii) −*x*, *y*−3/2, −*z*+1/2.
